# Combination of probenecid-sulphadoxine-pyrimethamine for intermittent preventive treatment in pregnancy

**DOI:** 10.1186/1475-2875-11-39

**Published:** 2012-02-09

**Authors:** Julie Gutman, S Patrick Kachur, Laurence Slutsker, Alexis Nzila, Theonest Mutabingwa

**Affiliations:** 1 Division of Parasitic Diseases & Malaria, Malaria Branch, 1600 Clifton Rd. NE, Mailstop A06, Atlanta, GA 30329, USA; 2 Center for Global Health, Centers for Disease Control and Prevention, 1600 Clifton Rd. NE, Mailstop D-69, Atlanta, GA 30329-4018, USA; 3 Department of Chemistry, King Fahd University of Petroleum and Minerals, PO Box 468, Dhahran, 31261, Saudi Arabia; 4 Department of Community Medicine, Hubert Kairuki Memorial University, Dar-es-Salaam, Tanzania

**Keywords:** Malaria, Sulphadoxine-pyrimethamine, Probenecid, Pregnancy

## Abstract

The antifolate sulphadoxine-pyrimethamine (SP) has been used in the intermittent prevention of malaria in pregnancy (IPTp). SP is an ideal choice for IPTp, however, as resistance of *Plasmodium falciparum *to SP increases, data are accumulating that SP may no longer provide benefit in areas of high-level resistance. Probenecid was initially used as an adjunctive therapy to increase the blood concentration of penicillin; it has since been used to augment concentrations of other drugs, including antifolates. The addition of probenecid has been shown to increase the treatment efficacy of SP against malaria, suggesting that the combination of probenecid plus SP may prolong the useful lifespan of SP as an effective agent for IPTp. Here, the literature on the pharmacokinetics, adverse reactions, interactions and available data on the use of these drugs in pregnancy is reviewed, and the possible utility of an SP-probenecid combination is discussed. This article concludes by calling for further research into this potentially useful combination.

## Background

### Decreasing efficacy of sulphadoxine-pyrimethamine for intermittent prevention of malaria in pregnancy

The antifolate sulphadoxine-pyrimethamine (SP) has been used in the intermittent prevention and treatment of malaria in pregnancy; in addition, it has been explored for intermittent preventive treatment (IPT) in infants and children [1-7]. SP is an ideal choice for intermittent preventive treatment in pregnancy (IPTp), as it is effective as a single dose given two to three times in pregnancy, with an interval of at least one month between doses. Data from Mali suggest that three doses are significantly more effective than two doses [8]. Resistance of *Plasmodium falciparum *to SP has been increasing, and its use is no longer recommended for treatment of malaria in Africa [9-11]. In some areas where SP is no longer an effective malaria therapy, IPTp with SP has been shown to be beneficial to HIV-uninfected pregnant women, possibly as a result of their pre-existing immunity [1]. Of concern, however, is a recent report from Muheza, an area of Tanzania with high level SP resistance, suggesting that its use for IPTp may exacerbate resistance [12]. In that study, SP-IPTp was associated with a 5.4% increase (*p *= 0.003) in the prevalence of parasitaemia in women who reported SP-IPTp use compared to those who did not. In addition, women who reported SP-IPTp use had both a higher prevalence of placental inflammation by histopathology and higher intensity of inflammation than women who did not report using SP-IPTp [12]. A subsequent study from the same area showed the lack of beneficial pregnancy outcomes from SP-IPTp [13]. Data from Malawi also show that the effectiveness of SP-IPTp has been decreasing over time and no longer appears to provide any benefit [14]. In view of these findings, it is critical to search for new agents for IPTp; mefloquine or azithromycin-based combinations (including azithromycin plus chloroquine) currently appear most promising [15]. While searching for alternatives to SP-IPTp, data from in vitro experiments have shown that probenecid can increase the activity of the anti-malarial antifolates [16,17]. Furthermore, malaria treatment studies in Nigerian children have shown that the addition of probenecid increases the treatment efficacy of SP [18,19]. Implicitly, the combination of probenecid plus SP may prolong the useful lifespan of SP as an effective agent for IPTp.

### Mechanism of action and development of resistance to SP

**Figure 1:**
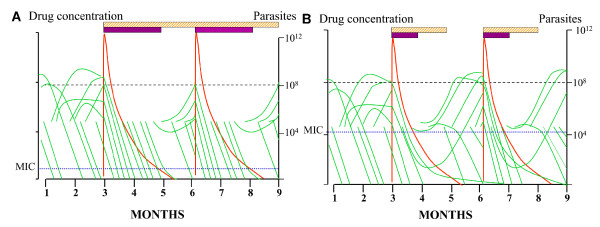
**Hypothetical parasite burden profiles during pregnancy with SP IPT in a high-transmission setting**. Entomological inoculation rate is about 50 infectious bites per person per year. Note that many infections self-cure (each infection is depicted as a green line). The hatched bars represent the duration of "suppressive prophylactic activity", and the solid bars represent the period during which parasite multiplication is suppressed (i.e. levels exceed the in vivo MIC). The horizontal dotted line at 10^8 ^parasites represents the level at which malaria can be detected on a blood film. (**A**) represents a drug-sensitive area; (**B**) represents a moderately resistant area. Reproduced from: White NJ (2005) Intermittent Presumptive Treatment for Malaria. PLoS Med 2(1): e3. doi:10.1371/journal.pmed.0020003

Sulphadoxine and pyrimethamine act synergistically to inhibit two steps in the folate synthesis pathway. Sulphadoxine inhibits the enzyme dihydropteroate synthetase (*dhps*) while pyrimethamine inhibits the enzyme dihydrofolate reductase (*dhfr*). Resistance develops in a step-wise manner due to a combination of mutations in these genes, with increasing numbers of mutations conferring increasing levels of resistance [20]. This results in an increased minimal inhibitory concentration (MIC), which translates to a decreased duration of prophylaxis because as drug levels fall, they reach the point at which they no longer suppress parasite replication sooner (Figure 1) [21]. The presence of the "quintuple mutant" consisting of the *dhfr *triple mutant (point mutations causing a Ser → Asn change at position 108, Asn → Ile at codon 51, and Cys → Arg at codon 59) and the *dhps *double mutant (due to point mutations which convert Ala → Gly at codon 437 and Lys → Glu at codon 540) has been most strongly associated with treatment failure [22], although host immunity still plays a significant role in determining drug efficacy [23]. Although theoretically, increasing the dose of SP could potentially overcome resistance to some degree, modelling suggests that increasing the dose of SP dose will only have a marginal benefit since highly resistant parasites will not be efficiently cleared by in vivo drug levels achievable within safety margins [24]. Furthermore, data in pregnant women indicate that there is significant variability in the effect of pregnancy on the disposition of SP, thus it was concluded that it is not possible to recommend an adjustment to the dose of SP to increase its efficacy [25].

### Rationale for use of probenecid

Probenecid was developed in 1950 to inhibit the renal tubular secretion of penicillin, increasing the blood concentration of the drug and decreasing the required dose [26,27]. Since its introduction, it has been found to inhibit the tubular secretion of other weak organic acids, such as p-aminohippuric acid, salicylic acid and uric acid, as well as other antibiotics (including cephalosporins), and anti-retrovirals (including oseltamavir and zidovudine). This characteristic of probenecid allows for decreased doses of medications to be used, thus limiting toxicity, especially with nephrotoxic agents [27-30]. Probenecid has been widely used as an uricosuric agent in the treatment of gout [26], and has a long history of use without serious toxicity [31]. It is widely available and inexpensive, costing approximately US$1 per 500 mg tablet.

Probenecid is a substrate for multi-drug resistance-associated protein (MRP) and the uric acid transporter [32] and is an inhibitor of both organic anion transporters 1 and 3 (OAT1 and OAT3) [33-35]. Probenecid increases plasma concentrations of the antifolate methotrexate by inhibiting drug efflux mediated by the MRP family of ATP-binding cassette transporters while at the same time inhibiting folate uptake [36]. This has been shown to reverse some forms of methotrexate resistance, and can increase the anti-cancer efficacy of antifolate drugs (methotrexate analogs) [36,37].

For treatment of *P. falciparum *infections, studies have clearly demonstrated that the addition of folate derivatives decreases the activity of antifolate drugs, both in vitro and in vivo [38-40]. Likewise, the lowering of folate concentration in vivo enhances the activity of antifolate anti-malarial agents [41]. This, taken together with the data from the studies of methotrexate, suggests that probenecid may be able to increase the efficacy of antifolates against resistant *Plasmodium *parasites by increasing the concentration of drug and limiting the uptake of folate. This has been demonstrated in vitro for the antifolates sulphadoxine, dapsone, pyrimethamine, and chlorcycloguanil [17]. In the presence of clinically achievable probenecid concentrations (50 μM or 14.268 mcg/ml), the activity of these antifolates was significantly increased. Increased activity ranged from two- to seven-fold, and in the case of resistant parasite isolates, reduced half maximal inhibitory concentrations (IC_50) _to levels seen in drug sensitive isolates [17]. These improvements in drug susceptibility correlated with reduced folate uptake in the presence of probenecid [17]. Probenecid has also been shown to reverse chloroquine resistance and increase piperaquine activity in vitro [17,42].

Probenecid has been shown to increase the efficacy of SP for the treatment of malaria in clinical trials in Nigerian children in an area with approximately 25% resistance to SP. In a study of 151 children under the age of 12 years, Sowunmi et al. showed that the addition of approximately 20-25 mg/kg/d of probenecid for three-days to standard SP dosing increased the efficacy of SP, as measured by the parasitological cure rate at day 14, compared to children who received SP alone (96.2% vs 83.5%, *p *= 0.02, n = 78 and 73, respectively) [19]. Although the cure rate in the SP-probenecid group remained higher at day 28, this was no longer statistically significant (79.4% vs 72.6%, *p *= 0.4) [19]. The combination of SP-probenecid was well tolerated in this small study and had no significant side effects [19]. Similar findings were reported from the same study population, with a 14-day cure rate of 100% in the SP-probenecid arm (n = 5) compared to 88% in the SP arm (n = 25) in one study and 94.8% (n = 39) compared to 76.4% (n = 34, *p *= 0.02) in another [18,43].

Although probenecid has been in use for over half a century, and has been recommended as part of the treatment of gonorrhea in pregnancy, very little clinical data exist on its safety in pregnancy. Furthermore, no clinical studies have been done on the pharmacokinetics of probenecid in pregnancy. Available evidence suggests that probenecid may potentiate the effect of SP and thus prolong its use for IPTp. However, data from pregnant women are needed to explore this potential. This review presents available data and highlights the knowledge gaps surrounding potential use of SP-probenecid combination as an effective agent for IPTp.

### Pharmacokinetics

#### Probenecid

Probenecid is packaged as 500 mg tablets. It is a weak acid (pKa 3.7) [44], and its oral bioavailability is > 90% [45]. In healthy adults, probenecid is 85-95% bound to plasma albumin and has a small apparent volume of distribution of 0.003-0.014 L/kg [44,46]. The maximum adult dose of probenecid is 3 g, and a single oral dose of 2 g (approximate 25 mg/Kg) yields peak plasma concentrations of 150-200 mcg/mL within four hours; concentrations above 50 mcg/mL are sustained for eight hours [31,47]. Following a 2 g dose, the half-life is 4-17 h; the half-life is dose-dependent, decreasing as the dose decreases to 500 mg [31]. Probenecid is metabolized in the liver via oxidation and glucuronidation and is primarily excreted in the urine (75-85%) [27]. No dosage adjustment is necessary for patients with mild renal failure (glomerular filtration rate greater than 50 mL/min); probenecid is not effective in severe renal failure and should be avoided [48,49]. No data are available on the pharmacokinetics of probenecid in pregnant women.

#### Sulphadoxine-pyrimethamine (SP)

SP is packaged as a fixed dose combination containing 500 mg sulphadoxine and 25 mg pyrimethamine and costs approximately US$1 per treatment (adult dosage is three tablets). In the non-pregnant population, both sulphadoxine and pyrimethamine have an oral bioavailability of > 90%, and are 85-90% protein bound [25]. The volume of distribution for sulphadoxine and pyrimethamine is 0.14 L/kg and 2.3 L/kg, respectively [50]. Oral administration of one tablet yields peak plasma levels of approximately 0.2 mg/L for pyrimethamine and approximately 60 mg/L for sulphadoxine at approximately four hours [50]. Both compounds have a very long half-life; the half-life of sulphadoxine is approximately 169 h (range 100-230 h), while that of pyrimethamine is approximately 111 h (range 54-148 h) [51]. Sulphadoxine is metabolized via glucuronidation and excreted primarily in urine, while pyrimethamine is metabolized to several unidentified metabolites but also excreted primarily in the urine [25,52].

Several studies have examined the pharmacokinetics of SP in pregnant women. All examined the standard dose for IPTp of three tablets (1500 mg of sulphadoxine and 75 mg of pyrimethamine). Nyunt et al. studied women in four African countries both during pregnancy and six to eight weeks post-partum, and found that, at Day 7 following administration of SP, there was an increased blood concentration of pyrimethamine, with an area under the curve (AUC) of 1,906 ng·day/ml during pregnancy vs 849 ng·day/ml postpartum, with geometric mean ratio of pharmacokinetic values between pregnancy and postpartum period 1.38 (1.22-1.56), *p*-value < 0.0001. In addition, they found decreased concentration of sulphadoxine with an AUC of 877 μg·day/ml during pregnancy versus 884 μg·day/ml postpartum, with geometric mean ratio of pharmacokinetic values between pregnancy and postpartum period 0.88 (0.80-0.96), *p*-value = 0.004, also on Day 7. However, there was significant variation in the exact pharmacokinetic parameters among the different study sites [25]. Findings from a study done in Kisumu, Kenya by Green et al., looking at levels of SP during pregnancy compared to eight-12 weeks post-partum, found similar results for sulphadoxine [53]. However, pyrimethamine pharmacokinetics were not significantly different in pregnant vs non-pregnant women [53]. In a third study conducted in Papua New Guinea where pregnant women were matched to non-pregnant controls, plasma concentrations of both sulphadoxine and pyrimethamine were found to be lower in the pregnant women compared to the non-pregnant controls (AUC of 22,315 vs 33,284 mg·h/l for sulphadoxine and 72,115 vs 106,065 μg·h/l for pyrimethamine; *p*-value < 0.001 for both) [54]. These varying results may be due to genetic differences in the women that contribute to differential drug metabolism, as well as to differences in body weight.

There are no data on how the combination of probenecid, sulphadoxine, and pyrimethamine affects pharmacokinetics or pharmacodynamics of the individual drugs. Further studies are needed to define these effects and to determine optimal dosing regimens. Given the significant differences in the half lives of SP and probenecid, there is also a need to optimize the probenecid regimen to maximize efficacy of SP while minimizing the frequency of administration to facilitate adequate compliance and adherence.

### Adverse events

#### Probenecid

Adverse reactions with probenecid are quite rare, occurring in approximately 0.1-0.3% of the general population, although the rate in pregnancy is unknown [55]. While rare, adverse reactions have been reported in almost all organ systems, including gastrointestinal, dermatologic, hematologic, renal, and immunologic (anaphylaxis) [26]. Isolated cases of aplastic anaemia, leukopenia, neutropenia, and thrombocytopenia have been reported [56]. Early reports suggested that probenecid may be associated with haemolysis in individuals with glucose-6-phosphatase dehydrogenase (G6PD) deficiency [57]. However, this has not been shown to be the case in subsequent studies [58,59]. Furthermore, recent reviews report that probenecid is not likely to cause haemolysis in patients with G6PD deficiency [60,61]. Probenecid has, however, been associated with immune mediated haemolytic anaemia in several case reports [62,63]. Nephrotic syndrome, which typically resolves on removal of drug, has been reported in a very small number of cases, with one case progressing to death [64-68]. There has also been a single case report of fatal haepatic necrosis [69]. Hypersensitivity reactions to probenecid in the general population may occur in as many as 2% to 4%. The incidence of probenecid hypersensitivity is much higher in HIV-infected patients ranging from 11% to 25% [70]. These reactions ranged from a mild rash that resolved with continued drug administration to more severe reactions consisting of a diffuse rash accompanied by constitutional symptoms, including fever and hypotension, leading to discontinuation of the drug [71-73]. There has been a single case report of anaphylactoid reaction after a single oral dose of probenecid [74].

#### Sulphadoxine-pyrimethamine (SP)

Despite a long list of potentially severe side effects associated with sulphonamides, the dose of SP used for IPTp is generally well tolerated [50,51]. Bone marrow suppression including agranulocytosis, aplastic anaemia, megaloblastic anaemia, thrombocytopenia, leukopenia, haemolytic anaemia, and eosinophilia may be seen. Haematologic adverse events are related to folic acid antagonism, and can generally be reversed with folinic acid [51]. Mild adverse reactions, such as nausea, vomiting, rash, pruritus, and fatigue have been reported in a small proportion (1-2%) of patients following a single dose of SP [1,75-77]. Parise et al. reported an increased incidence of adverse events in women with HIV, but this was not confirmed in a study by Filler et al. [75,76]. Severe adverse events are rare [1]. Cutaneous hypersensitivity reactions (i.e. Stevens-Johnson syndrome and toxic epidermal necrolysis) are the most common severe adverse events. In studies of travellers taking weekly SP for malaria prophylaxis, the rate of cutaneous hypersensitivity reactions has been reported to be approximately one per 5,000-8,000, with one fatality per 11,000-25,000 [78-80]. The crude rate of cutaneous hypersensitivity reaction was reported to be approximately 1.2 per 100,000 exposures to SP in passive surveillance data from 22 health facilities in Blantyre, Malawi [81]. In that study, the rate of cutaneous hypersensitivity in HIV-infected individuals was approximately four times the crude rate, at 4.9 cases per 100,000 [81]. In a study comparing IPTp with standard SP to SP plus azithromycin, Luntamo et al. reported 14 maternal severe adverse events, including one maternal death, among 1,320 women (1%). However, the majority of these were considered to be unrelated to the study drugs [82]. It is unclear whether any of the reported adverse events were severe cutaneous reactions. In a systematic review of IPTp-SP, ter Kuile et al. report a total of 21 maternal deaths among 11,379 doses of SP given to 4,911 women; nine of these occurred in women receiving placebo [1]. Only one of these deaths was reported to be due to a severe cutaneous hypersensitivity reaction, and it occurred in an HIV-positive woman three weeks after the dose of SP [1,77]. A more comprehensive review of cutaneous hypersensitivity reactions was conducted by Peters et al. [51]. Other, less common, severe reactions that have been seen with SP use include liver toxicity (e.g. cholestatic hepatotoxicity and fulminant hepatic necrosis), fever, and respiratory problems such as hypersensitivity pneumonitis [51]. Patients with G6PD deficiency can safely receive SP-IPTp or malaria treatment with SP [51,83].

### Pregnancy

#### Probenecid

Probenecid is known to cross the placental barrier and appears in cord blood [48]. There are limited data on the use of probenecid in pregnancy, with approximately 460 cases described in the literature (Table 1). In most of these cases, no data are provided on the outcome of pregnancy. Of the few studies that describe foetal outcomes, none shows a statistically significant increase in foetal adverse events in infants exposed to probenecid compared to controls [84], although the small sample size of most of these studies is a major limitation to effectively evaluating infant outcomes. Due to the paucity of data, the US Food and Drug Administration classifies probenecid as Pregnancy Category B. However, the data available do not suggest any evidence of teratogenic effects of probenecid when used in pregnancy [48,55].

**Table 1:** Reports of probenecid use during pregnancy

Author	Study	N	Treatment	Timing of Treatment	SAEs	Infant Outcome
Cavenee et al. [84]	Treatment of gonorrhoea in pregnancy	123	Amoxicillin 3 gm + probenecid 1 gm	64 patients (25%) treated in 1st trimester of pregnancy	None, one woman reported vomiting several hours after taking the drug	71 infants were evaluated, 14 treated < 14 week, 1 major malformation (7%), 4 minor malformations (29%); 57 > 14 week, 0 major and 10 minor malformations (18%), overall 1% major and 20% minor malformations, not statistically different from other groups

Adelson et al. [85]	Treatment of urinary tract infections in pregnancy	98	Ampicillin 3.5 gm + probenecid 1 gm	Not stated	3 patients developed candidal vulvo-vaginitis, 3 patients developed diarrhoea	Not stated

Brown et al. [86]	Renal clearance of oestrogen in pregnancy	9	Probenecid 2.5 gm	Last 6 weeks of pregnancy	None reported	No foetal deaths or stillbirths

Lee et al. [87]	Gout and pregnancy	1	Probenecid 1 gm to 3 gm daily throughout pregnancy	Throughout pregnancy	Anaemia thought related to renal disease	Healthy infant

Weingold et al. [88]	Gout and pregnancy	1	Colchicine and probenecid	Throughout 1st pregnancy and for the first 14 weeks of 2^nd ^pregnancy	Mild anaemia	1st pregnancy: infant died on DOL4 due to hyaline membrane disease 2^nd ^pregnancy: healthy infant

Goodrich [55]	Gonorrhoea and pregnancy	163	Penicillin G 4.8 × 10^6 ^U (n = 158) or ampicillin (n = 5) + probenecid 1 gm	Not stated	None reported	Not stated

Schackis [89]	Hype-ruricemia and pre-eclampsia	20*	Probenecid 250 mg twice daily for 7 days	26-32 weeks gestation	No side-effects to probenecid were recorded	There were 3 intrauterine foetal deaths: 1 from an abruptio placenta (> 1 kg) in the probenecid group and 2 from suspected placental insufficiency (both < 1 kg): 1 each in the probenecid and placebo group.

Czaczkes et al. [90]	Pre-eclampsia, eclampsia	18†	Probenecid 0.5 gm 3 times daily for 5-6 days	Not stated	None reported	Not stated

*20 women treated and an additional 20 untreated controls

†15 women with pre-eclampsia + 3 pregnant controls without pre-eclampsia

### Sulphadoxine-pyrimethamine

SP is classified by the US Food and Drug Administration as belonging to Pregnancy Category C. Pyrimethamine is teratogenic at doses well above the standard human dose in rats, hamsters and miniature pigs; effects including foetal re-absorption, cleft palate, bradygnathia, oligodactyly, and microphthalmia have been seen [50]. Sulphadoxine is also teratogenic in rats [50]. In rabbits, no teratogenic effects were noted at oral doses as high as 20 mg/kg pyrimethamine plus 400 mg/kg sulphadoxine [50]. One large study in humans looking at the effect of folic acid antagonists found an increased risk of malformations, including cardiovascular defects, oral clefts, and urinary tract defects, with exposure in the first trimester, but not in subsequent trimesters [91]. However, several large studies of SP-IPTp, including several randomized controlled trials, have not found an association between the use of SP and congenital anomalies [67,68]. Therefore, despite data suggesting teratogenicity in certain animal models, there is a large body of evidence suggesting that SP-IPTp is safe for use in humans during the second and third trimesters. Of note, probenecid in combination with SP would potentiate the antifolate effect of SP [36], with the potential danger of reducing the folates available to the growing foetus. Therefore, should this combination be adopted, careful monitoring is suggested to ensure that the combination does not have harmful effects [13].

Both pyrimethamine and sulphadoxine cross the placental barrier and also pass into breast milk [50]. Sulphonamides could theoretically increase the risk of kernicterus in the infant if given to a pregnant woman near term, due to the ability of sulphonamides to displace unconjugated bilirubin from albumin [51]. However, this has not actually been seen in clinical trials of IPTp, clinical treatment of cases of malaria or in the treatment of congenital toxoplasmosis (which uses pyrimethamine, sulphadiazine and folinic acid) [51,91].

### Drug interactions

#### Probenecid

Probenecid is a ucosuric agent that inhibits the tubular secretion of organic anion derivatives. These organic anions (such penicillin and analogs), which are primarily derivatives of carboxylic acid (DCA), are secreted in the kidney by organic anion transporters (OATs). Probeneicd, which is also a DCA, interacts with these OATs, leading to a decrease in DCA secretion [92,93]. Thus, the mechanism of action of probenecid depends on the inhibition of OATs, reducing the secretion of DCA. Most of the drugs reported in the list below are organic anion compounds that are secreted through OATs, hence their interaction with probenecid. In the next section, we detail specific probenecid-drug interaction.

### List of drugs whose concentration or effects are increased by probenecid

• **Antibacterials**: Penicillins, Cephalosporins, Carbapenems, Quinolones, Dapsone, Rifampin, Tazobactam

• **Antivirals**: Ganciclovir, Valganciclovir, Oseltamavir, Peramavir

• **Antiretrovirals**: Zalcitabine, Zidovudine

• Methotrexate

• Acetaminophen

• **Non-steroidal anti-inflammatory drugs**: Indomethacin, Ketoprofen, Ketorolac, Naproxen, Zomepirac

• **Benzodiazepines**: Lorazepam, Midazolam, Nitrazepam

• Chlorothiazide (thiazide)

• **Loop Diuretics**: Bumetanide, Furosemide

• Mycophenolate

• **Theophylline Derivatives**: Dyphylline, Enprofylline but NOT Aminophylline or Theophylline

• **Sulphonylureas: **Glimepiride, Glyburide

• Nitrofurantoin

• Apazone

• Entacapone

• Famotidine

• Pemetrexed

• Pralatrexate

• Sodium Phenylacetate, Sodium Benzoate

Probenecid is antagonized by both salicylates and pyrazinamide [31]. Allopurinol decreases the serum concentration of probenecid while probenecid increases that of allopurinol. However, the clinical significance of this is probably minimal [31]. Probenecid decreases the natriuretic effect of piretanide [31]. Probenecid increases the metabolism of phenprocoumon, leading to decreased AUC [94]. Probenecid has a nephroprotective effect when co-administered with cidofovir, but without significantly altering plasma blood levels [94]. It is a weak inhibitor of CYP2C19 and blocks the renal transport of many compounds including many classes of antibiotics, antivirals, and NSAIDs leading to an increase in their mean plasma elimination half-life that can lead to increased plasma concentrations [31]. In some cases, this may increase the potential for toxicity.

#### Sulphadoxine-pyrimethamine

Neither sulphadoxine nor pyrimethine, nor their primary metabolites, are DCA [95,96], thus the mechanisms of secretion of these two drugs are not likely to involve OATs. Therefore, their pharmacokinetics would not be affected by probenecid. In support of this hypothesis, the clinical evaluation of probenecid with SP did not indicate any increase in sulfadoxine or pyrimethamine toxicity [19]. However, further investigations need to be carried out to establish the pharmacokinetics, safety, and efficacy of probenecid + SP, before this combination could be used routinely for IPTp.

**SP **should not be used in combination with other antifolates (i.e. trimethoprim), sulphonamides, or other agents associated with myelosuppression, as this can increase the risk of bone marrow suppression (Table 2). The concurrent use of gold salts and anti-malarial agents is also contraindicated due to an increased risk of blood dyscrasias. Sulphadoxine decreases the serum concentration of cyclosporine while enhancing its nephrotoxic effect. Potassium P-aminobenzoate and its derivatives (Procaine, benzocaine, and tetracaine) can decrease the therapeutic effect of sulphadoxine, while folate derivatives can decrease the therapeutic effect of pyrimethamine. The concomitant administration of antacids or absorbent anti-diarrhoeals may significantly reduce the bioavailability of pyrimethamine by reducing its absorption. Pyrimethamine inhibits CYP2C9 and CYP 2D6, and may therefore interact with other compounds, which are metabolized via these pathways (Table 3).

**Table 2:** Drugs that interact with sulphadoxine

Drug	Effect of Interaction
**Phenytoin**	Serum hydantoin levels and risk of toxicity may be increased due to inhibition of hepatic metabolism by sulphadoxine

**Sulphonylureas **(Glimepiride, Glyburide)	The hypoglycemic potential of the sulphonylureas may be increased; the mechanism of this interaction is unknown.

**Vitamin K antagonists **(Coumadin, anisindione)	Co-administration with a sulphonamide may increase the plasma concentrations and hypoprothrombinemic effects of coumarin anticoagulants

**Gold salts**	Increase the risk of blood dyscrasias

**Cyclosporine**	Sulphadoxine decreases the serum concentration of cyclosporine while enhancing its nephrotoxic effect

**Table 3:** Drugs that interact with pyrimethamine

Drug	Effect of Interaction
**Gold salts**	Increased risk of blood dyscrasias

**Methotrexate**	Increased risk of bone marrow suppression

**Dapsone**	Folate antagonists may increase the likelihood of adverse hematologic reactions (e.g., agranulocytosis, anaemia)

**Carvedilol**	Possible for increased serum concentration of the S-carvedilol enantiomer

**Phenothiazines**	Anti-malarial agents may increase the serum concentration

**Fesoterodine**	Serum concentrations of the active metabolite may be increased

**Nebivolol**	Serum concentration may be increased

**Zidovudine, abacavir, lamivudine**	Increased risk of bone marrow suppression

**Tamoxifen**	Metabolism of Tamoxifen to the active metabolites may be decreased

**Lorazepam**	Increases risk of elevated liver function tests

**Codeine**	CYP2D6 inhibitors may prevent the metabolic conversion of codeine to its active metabolite morphine, diminishing its therapeutic effect

**Tramadol**	CYP2D6 inhibitors may prevent the metabolic conversion to the active metabolite, diminishing its therapeutic effect

**Antacids**	Reduce the absorption of pyrimethamine (decreased bioavailability)

SP-IPTp works both through clearance of existing asymptomatic infections as well as prophylactically by preventing new infections [1]. The prophylactic effect of SP is believed to be of primary importance [1]. Due to the long half-life of SP, its parasitocidal drug levels are sustained for weeks in the case of sensitive parasites, providing a prolonged period of prophylaxis. As parasite resistance to SP increases, the period of prophylactic efficacy becomes shorter [21]. Even if probenecid can increase the concentration of SP, given the very short half-life of probenecid (less than 20 h) compared to SP (approximately 100 h), this effect is unlikely to be sustained. Therefore, while the addition of a single dose of probenecid to SP may be useful for treatment, it is unclear to what extent it will provide a prolonged period of prophylaxis in the context of IPTp. Multiple doses of probenecid may be needed to provide a sustained benefit of SP-IPTp. However, a multiple-dose regimen would be less ideal as compliance with such a regimen is likely to be low.

## Conclusions

The antifolate SP is widely used for IPTp; it is the only drug currently recommended for IPTp by WHO and has been adopted as national policy in 37 countries worldwide, 33 of which are in the sub-Saharan region [97,98]. However, resistance of malaria parasites to SP has been increasing, and there is accumulating evidence from eastern Africa that the efficacy of SP-IPTp is declining. Therefore, new drugs and strategies are urgently needed. As malaria transmission declines, the risk associated with administering a drug to all pregnant women will no longer outweigh the potential benefit associated with preventing malaria, and it is likely that IPTp will be abandoned. In the interim, in vitro and in vivo data suggest that combining probenecid with SP significantly increases the efficacy of SP, and might therefore be a useful strategy in HIV-uninfected women until new drugs for IPTp are evaluated and deployed [13-16], although this combination may not be sufficiently efficacious in areas with a high degree of resistance. Although probenecid has been in use for many decades, very few data exist on its use in pregnancy; however, the available data suggests that it is safe. Prior to recommending this combination for widespread use, additional information is needed on its safety and pharmacokinetics in pregnancy, and given the difference in the half-lives of SP and probenecid, data are needed to determine the optimal dosing schedule. In addition, studies are needed to determine whether the increased dose of SP achieved by this combination improves the efficacy against partially resistant parasites. Mathematical modelling may be able to assist in this determination. Overall, available data on efficacy, safety and cost for these drugs necessitates further research into their combination for use in IPTp [48,55].

## Abbreviations

SP: Sulphadoxine-pyrimethamine; IPTp: Intermittent preventive treatment in pregnancy; *dhps: *Dihydropteroate synthetase; *dhfr: *Dihydrofolate reductase; G6PD: Glucose-6-phosphatase dehydrogenase; MRP: Multidrug resistance-associated protein; AUC: Area under the curve; OAT: Organic anion transporter; DCA: Derivatives of carboxylic acid

## Competing interests

The authors declare that they have no competing interests.

## Authors' contributions

TK and AN were responsible for the concept of the manuscript. JG was responsible for the acquisition of data, interpretation of the data and writing the manuscript. SPK, LS, AN, and TK contributed to interpretation of the data and critically revised the paper. All authors have seen and approved the final version.

## References

[B1] ter Kuile FO van Eijk AM Filler SJ Effect of sulfadoxine-pyrimethamine resistance on the efficacy of intermittent preventive therapy for malaria control during pregnancy JAMA 2007 297 2603 2616 10.1001/jama.297.23.2603 17579229

[B2] Aponte JJ Schellenberg D Egan A Breckenridge A Carneiro I Critchley J Danquah I Dodoo A Kobbe R Lell B May J Premji Z Sanz S Sevene E Soulaymani-Becheikh R Winstanley P Adjei S Anemana S Chandramohan D Issifou S Mockenhaupt F Owusu-Agyei S Greenwood B Grobusch MP Kremsner PG Macete E Mshinda H Newman RD Slutsker L Tanner M Efficacy and safety of intermittent preventive treatment with sulfadoxine-pyrimethamine for malaria in African infants: a pooled analysis of six randomised, placebo-controlled trials Lancet 2009 374 1533 1542 10.1016/S0140-6736(09)61258-7 19765816

[B3] Dicko A Sagara I Sissoko M Guindo O Diallo A Kone M Toure O Sacko M Doumbo O Impact of intermittent preventive treatment with sulphadoxine-pyrimethamine targeting the transmission season on the incidence of clinical malaria in children in Mali Malar J 2008 7 123 10.1186/1475-2875-7-123 18611271 PMC2500037

[B4] Thwing J Eisele TP Steketee RW Protective efficacy of malaria case management and intermittent preventive treatment for preventing malaria mortality in children: a systematic review for the Lives Saved Tool BMC Publ Health 2011 11 Suppl 3 S14 10.1186/1471-2458-11-S3-S14 PMC3231887 21501431

[B5] Wilson A IPTc Taskforce A systematic review and meta-analysis of the efficacy and safety of intermittent preventive treatment of malaria in children (IPTc) PLoS One 2011 6 e16976 10.1371/journal.pone.0016976 21340029 PMC3038871

[B6] Greenwood B Review: Intermittent preventive treatment--a new approach to the prevention of malaria in children in areas with seasonal malaria transmission Trop Med Int Health 2006 11 983 91 10.1111/j.1365-3156.2006.01657.x 16827699

[B7] Gosling RD Carneiro I Chandramohan D Intermittent preventive treatment of malaria in infants: how does it work and where will it work? Trop Med Int Health 2009 14 1003 1010 10.1111/j.1365-3156.2009.02303.x 19558374

[B8] Maiga OM Kayentao K Traoré BT Djimde A Traoré B Diallo M Ongoiba A Doumtabé D Doumbo S Traoré MS Dara A Guindo O Karim DM Coulibaly S Bougoudogo F Ter Kuile FO Danis M Doumbo OK Superiority of 3 over 2 doses of intermittent preventive treatment with sulfadoxine-pyrimethamine for the prevention of malaria during pregnancy in Mali: a randomized controlled trial Clin Infect Dis 2011 53 215 223 10.1093/cid/cir374 21765069

[B9] World Health Organization Susceptibility of *Plasmodium falciparum *to antimalarial drugs: Report on global monitoring: 1996-2004 Technical document WHO/HTM/MAL/20051103 Geneva: World Health Organization 2005

[B10] Gesase S Gosling RD Hashim R Ord R Naidoo I Madebe R Mosha JF Joho A Mandia V Mrema H Mapunda E Savael Z Lemnge M Mosha FW Greenwood B Roper C Chandramohan D High resistance of *Plasmodium falciparu *to sulphadoxine/pyrimethamine in Northern Tanzania and the emergence of dhps resistance mutation at codon 581 PLoS One 2009 4 e4569 10.1371/journal.pone.0004569 19238219 PMC2644264

[B11] World Health Organization Guidelines for the treatment of malaria 2010 2 Geneva: WHO 25473692

[B12] Harrington WE Mutabingwa TK Muehlenbachs A Sorensen B Bolla MC Fried M Duffy PE Competitive facilitation of drug-resistant *Plasmodium falciparu *malaria parasites in pregnant women who receive preventive treatment Proc Natl Acad Sci USA 2009 106 9027 9032 10.1073/pnas.0901415106 19451638 PMC2690058

[B13] Harrington WE Mutabingwa TK Kabyemela E Fried M Duffy PE Intermittent Treatment to prevent pregnancy malaria does not confer benefit in an area of widespread drug resistance Clin Infect Dis 2011 53 224 230 10.1093/cid/cir376 21765070 PMC3202321

[B14] Feng G Simpson JA Chaluluka E Molyneux ME Rogerson SJ Decreasing burden of malaria in pregnancy in Malawian women and its relationship to use of intermittent preventive therapy or bed nets PLoS One 2010 5 e12012 10.1371/journal.pone.0012012 20700457 PMC2917365

[B15] Gosling RD Cairns ME Chico RM Chandramohan D Intermittent preventive treatment against malaria: an update Expert Rev Anti Infect Ther 2010 8 589 606 10.1586/eri.10.36 20455687

[B16] Nzila AM Kokwaro G Winstanley PA Marsh K Ward SA Therapeutic potential of folate uptake inhibition in *Plasmodium falciparu* Trends Parasitol 2004 20 109 112 10.1016/j.pt.2003.12.005 16676416

[B17] Nzila A Mberu E Bray P Kokwaro G Winstanley P Marsh K Ward S Chemosensitization of *Plasmodium falciparu *by Probenecid In Vitro Antimicrob Agents Chemother 2003 47 2108 2112 10.1128/AAC.47.7.2108-2112.2003 12821454 PMC161864

[B18] Sowunmi A Adedeji AA Fateye BA Fehintola FA Comparative effects of pyrimethamine-sulfadoxine, with and without probenecid, on *Plasmodium falciparu *gametocytes in children with acute, uncomplicated malaria Ann Trop Med Parasitol 2004 98 873 878 10.1179/000349804X3243 15667719

[B19] Sowunmi A Fehintola FA Adedeji AA Gbotosho GO Falade CO Tambo E Fateye BA Happi TC Oduola AMJ Open randomized study of pyrimethamine-sulphadoxine vs. pyrimethamine-sulphadoxine plus probenecid for the treatment of uncomplicated Plasmodium falciparum malaria in children Trop Med Int Health 2004 9 606 614 10.1111/j.1365-3156.2004.01233.x 15117306

[B20] Mita T Tanabe K Kita K Spread and evolution of *Plasmodium falciparu *drug resistance Parasitol Int 2009 58 201 209 10.1016/j.parint.2009.04.004 19393762

[B21] White NJ Intermittent Presumptive Treatment for Malaria PLoS Med 2005 2 e3 10.1371/journal.pmed.0020003 15696210 PMC545196

[B22] Kublin J Dzinjalamala F Kamwendo D Malkin E Cortese J Martino L Mukadam R Rogerson S Lescano A Molyneux M Winstanley PA Chimpeni P Taylor TE Plowe CV Molecular markers for failure of sulfadoxine-pyrimethamine and chlorproguanil-dapsone treatment of *Plasmodium falciparu *malaria J Infect Dis 2002 185 380 388 10.1086/338566 11807721

[B23] Gregson A Plowe CV Mechanisms of resistance of malaria parasites to antifolates Pharmacol Rev 2005 57 117 145 10.1124/pr.57.1.4 15734729

[B24] Watkins WM Mberu EK Winstanley PA Plowe CV The efficacy of antifolate antimalarial combinations in Africa: a predictive model based on pharmacodynamic and pharmacokinetic analyses Parasitol Today 1997 13 459 464 10.1016/S0169-4758(97)01124-1 15275132

[B25] Nyunt MM Adam I Kayentao K van Dijk J Thuma P Mauff K Little F Cassam Y Guirou E Traore B Doumbo O Sullivan D Smith P Barnes KI Pharmacokinetics of sulfadoxine and pyrimethamine in intermittent preventive treatment of malaria in pregnancy Clin Pharmacol Ther 2009 87 226 234 19776738 10.1038/clpt.2009.177

[B26] Boger WP Strickland SC PROBENECID (BENEMID) Its Uses and Side-Effects in 2,502 Patients AMA Arch Intern Med 1955 95 83 92 10.1001/archinte.1955.00250070099012 13217507

[B27] Cunningham R Israili ZH Dayton PG Clinical pharmacokinetics of probenecid Clin Pharmacokinet 1981 6 135 151 10.2165/00003088-198106020-00004 7011657

[B28] Holodniy M Penzak SR Straight TM Davey RT Lee KK Goetz MB Raisch DW Cunningham F Lin ET Olivo N Pharmacokinetics and tolerability of oseltamivir combined with probenecid Antimicrob Agents Chemother 2008 52 3013 3021 10.1128/AAC.00047-08 18559644 PMC2533494

[B29] Rayner CR Chanu P Gieschke R Boak LM Jonsson EN Population pharmacokinetics of oseltamivir when coadministered with probenecid J Clin Pharmacol 2008 48 935 947 10.1177/0091270008320317 18524996

[B30] Wolf DL Rodriguez CA Mucci M Ingrosso A Duncan BA Nickens DJ Pharmacokinetics and renal effects of cidofovir with a reduced dose of probenecid in HIV-infected patients with cytomegalovirus retinitis J Clin Pharmacol 2003 43 43 51 10.1177/0091270002239705 12520627

[B31] Probenecid Oral http://www.medscape.com/druginfo/dosage?drugid=8697&drugname=probenecid+Oral&monotype=default Accessed March 9, 2011

[B32] Roch-Ramel F Renal transport of organic anions Curr Opin Nephrol Hypertens 1998 7 517 524 10.1097/00041552-199809000-00006 9818198

[B33] Kusuhara H Sekine T Utsunomiya-Tate N Tsuda M Kojima R Cha SH Sugiyama Y Kanai Y Endou H Molecular cloning and characterization of a new multispecific organic anion transporter from rat brain J Biol Chem 1999 274 13675 13680 10.1074/jbc.274.19.13675 10224140

[B34] Sekine T Watanabe N Hosoyamada M Kanai Y Endou H Expression cloning and characterization of a novel multispecific organic anion transporter J Biol Chem 1997 272 18526 18529 10.1074/jbc.272.30.18526 9228014

[B35] Uwai Y Okuda M Takami K Hashimoto Y Inui K Functional characterization of the rat multispecific organic anion transporter OAT1 mediating basolateral uptake of anionic drugs in the kidney FEBS Lett 1998 438 321 324 10.1016/S0014-5793(98)01328-3 9827570

[B36] Sirotnak FM Wendel HG Bornmann WGB Tong WP Miller VA Scher HI Kris MG Co-administration of Probenecid, an inhibitor of a cMOAT/MRP- like plasma membrane ATPase, greatly enhanced the efficacy of a new 10-deazaaminopterin against human solid tumors in vivo Clin Cancer Res 2000 6 3705 3712 10999764

[B37] Bertino JR Goker E Gorlick R Li WW Banerjee D Resistance mechanisms to methotrexate in tumors Oncologist 1996 1 223 226 10387992

[B38] Watkins WM Sixsmith DG Chulay JD Spencer HC Antagonism of sulfadoxine and pyrimethamine antimalarial activity in vitro by p-aminobenzoic acid, p-aminobenzoylglutamic acid and folic acid Mol Biochem Parasitol 1985 14 55 61 10.1016/0166-6851(85)90105-7 3885030

[B39] Wang P Sims PF Hyde JE A modified in vitro sulfadoxine susceptibility assay for *Plasmodium falciparu *suitable for investigating Fansidar resistance Parasitology 1997 115 Pt 3 223 230 9300459 10.1017/s0031182097001431

[B40] Kinyanjui SM Mberu EK Winstanley PA Jacobus DP Watkins WM The antimalarial triazine WR99210 and the prodrug PS-15: folate reversal of *in vitr *activity against *Plasmodium falciparu *and a non- antifolate mode of action of the prodrug AmJTrop Med Hyg 1999 60 943 947 10.4269/ajtmh.1999.60.943 10403325

[B41] van Hensbroek MB Morris-Jones S Meisner S Jaffar S Bayo L Dackour R Phillips C Greenwood BM Iron, but not folic acid, combined with effective antimalarial therapy promotes haematological recovery in African children after acute falciparum malaria Trans R Soc Trop Med Hyg 1995 89 672 676 10.1016/0035-9203(95)90438-7 8594693

[B42] Kiara SM Okombo J Masseno V Mwai L Ochola I Borrmann S Nzila A In vitro activity of antifolate and polymorphism in dihydrofolate reductase of *Plasmodium falciparum *isolates from Kenyan coast: Emergence of parasites with Ile-164-Leu mutation Antimicrob Agents Chemother 2009 53 3793 3798 10.1128/AAC.00308-09 19528269 PMC2737895

[B43] Sowunmi A Adedeji AA Fateye BA Babalola CP Plasmodium falciparum hyperparasitaemia in children. Risk factors, treatment outcomes, and gametocytaemia following treatment Parasite 2004 11 317 323 15490757 10.1051/parasite/2004113317

[B44] Ilett KF Hackett LP Ingle B Bretz PJ Transfer of probenecid and cephalexin into breast milk Ann Pharmacother 2006 40 986 989 10.1345/aph.1G580 16551765

[B45] Anderson R Gambertoglio JG Schrier RW Clinical use of drugs in renal failure 1976 Springfield, Il: Charles C Thomas

[B46] Weber A de Groot R Ramsey B Williams-Warren J Smith A Probenecid pharmacokinetics in cystic fibrosis Dev Pharmacol Ther 1991 16 7 12 1879255

[B47] Selen A Amidon GL Welling PG Pharmacokinetics of probenecid following oral doses to human volunteers J Pharm Sci 1982 71 1238 1242 10.1002/jps.2600711114 7175716

[B48] Product Information: Benemid(R), probenecid tablets: Merck & Company 1998 West Point, PA

[B49] Bennett W Aronoff GR Golper TA Morrison G Singer I Brater DC Drug prescribing in renal failure 1987 Philadelphia: American College of Physicians 10.1016/s0272-6386(83)80060-2 6356890

[B50] Fansidar http://dailymed.nlm.nih.gov/dailymed/drugInfo.cfm?id=522&CFID=67813412&CFTOKEN=75538191fb984739-503250B9-9088-62D0-8FF3FAD9D8ECF54A&jsessionid=ca303b8ac4d678123f51 Accessed March 9, 2011

[B51] Peters PJ Thigpen MC Parise ME Newman RD Safety and toxicity of sulfadoxine/pyrimethamine: implications for malaria prevention in pregnancy using intermittent preventive treatment Drug Saf 2007 30 481 501 10.2165/00002018-200730060-00003 17536875

[B52] Monograph - Pyrimethamine, Sulfadoxine and Pyrimethamine http://www.medscape.com/druginfo/monograph?cid=med&drugid=5911&drugname=pyrimethamine+Oral&monotype=monograph&secid=10 Accessed March 9, 2011

[B53] Green MD van Eijk AM van ter Kuile FO Ayisi JG Parise ME Kager PA Nahlen BL Steketee R Nettey H Pharmacokinetics of sulfadoxine-pyrimethamine in HIV-infected and uninfected pregnant women in Western Kenya J Inf Dis 2007 196 1403 1408 10.1086/522632 17922406

[B54] Karunajeewa HA Salman S Mueller I Baiwog F Gomorrai S Law I Page-Sharp M Rogerson S Siba P Ilett KF Davis TM Pharmacokinetic properties of sulfadoxine-pyrimethamine in pregnant women Antimicrob Agents Chemother 2009 53 4368 4376 10.1128/AAC.00335-09 19620325 PMC2764169

[B55] Goodrich JT Treatment of gonorrhea in pregnancy Sex Transm Dis 1979 6 2 168 173 10.1097/00007435-197904000-00023 158835

[B56] Swanson M Cook R Drugs, chemicals and blood dyscrasias 1977 Hamilton, Il: Drug Intell Publications

[B57] Marks PA Banks J Drug induced hemolytic anemias associated with glucose-6-phosphate dehydrogenase deficiency: a genetically heterogenous trait Ann N Y Acad Sci 1965 123 198 206 14329202 10.1111/j.1749-6632.1965.tb12257.x

[B58] Chan T Todd D Tso SC Drug-induced haemolysis in glucose-6-phosphate dehydrogenase deficiency BMJ 1976 2 1227 1229 10.1136/bmj.2.6046.1227 990860 PMC1689719

[B59] Zail S Charlton RW Bothwell TH The hemolytic effect of certain drugs in Bantu subjects with a deficiency of glucose 6 phosphate dehydrogenase S Afr Med J 1972 27 95 99

[B60] Cappellini MD Fiorelli G Glucose-6-phosphate dehydrogenase deficiency Lancet 2008 371 64 74 10.1016/S0140-6736(08)60073-2 18177777

[B61] Youngster I Arcavi L Schechmaster R Akayzen Y Popliski H Shimonov J Beig S Berkovitch M Medications and glucose-6-phosphate dehydrogenase deficiency: an evidence-based review Drug Saf 2010 33 713 726 10.2165/11536520-000000000-00000 20701405

[B62] Sosler S Behzad O Garratty G Lee CL Postoway N Khomo O Immune hemolytic anemia associated with probenecid Am J Clin Pathol 1985 84 391 394 4036870 10.1093/ajcp/84.3.391

[B63] Kickler T Buck S Ness P Shirey RS Sholar PW Probenecid induced immune hemolytic anemia J Rheumatol 1986 13 208 209 3701734

[B64] Ferris TF Morgan WS Levitin H Nephrotic syndrome caused by probenecid NEJM 1961 265 381 383 10.1056/NEJM196108242650807 13699173

[B65] Hertz P Yager H Richardson JA Probenecid-induced nephritic syndrome Arch Pathol 1972 94 241 243 5051645

[B66] Izzedine H Brocheriou I Becart J Deray G Probenecid-induced membranous nephropathy Nephrol Dial Transplant 2007 22 2405 2406 17510101 10.1093/ndt/gfl798

[B67] Scott J O'Brien PK Probenecid, nephrotic syndrome, and renal failure Ann Rheum Dis 1968 27 249 252 10.1136/ard.27.3.249 5655316 PMC1031103

[B68] Sokol A Bashner MH Okun R Nephrotic syndrome caused by probenecid JAMA 1967 199 43 44 10.1001/jama.1967.03120010087026 6071127

[B69] Reynolds ES Schlant RC Gonick HC Dammin GJ Fatal massive necrosis of the liver as a manifestation of hypersensitivity to probenecid NEJM 1957 256 592 596 10.1056/NEJM195703282561304 13451901

[B70] Myers K Katial RK Engler RJ Probenecid Hypersensitivity in AIDS: a Case Report Ann Allergy Asthma Immunol 1998 80 416 418 10.1016/S1081-1206(10)62994-8 9609613

[B71] Lalezari JP Drew WL Glutzer E James C Miner D Flaherty J Fisher PE Cundy K Hannigan J Martin JC Jaffe HS (S)-1-[3-Hydroxy-2-(phosphonylmethoxy)propyl]cytosine (Cidofovir): results of a phase i/ii study of a novel antiviral nucleotide analogue J Inf Dis 1995 171 788 796 10.1093/infdis/171.4.788 7706804

[B72] Petty B Kornhauser D Lietman P Zidovudine with probenecid: a warning Lancet 1990 335 1044 1045 1970102 10.1016/0140-6736(90)91116-r

[B73] Polis MA Spooner KM Baird BF Manischewitz JF Jaffe HS Fisher PE Falloon J Davey RT Jr Kovacs JA Walker RE Anticytomegaloviral activity and safety of cidofovir in patients with human immunodeficiency virus infection and cytomegalovirus viruria Antimicrob Agents Chemother 1995 39 882 886 7785989 10.1128/aac.39.4.882 PMC162647

[B74] Hillecke N Acute anaphylactoid reaction to probenecid JAMA 1965 193 116 10.1001/jama.1965.03090090046018 14328478

[B75] Parise ME Ayisi JG Nahlen BL Schultz LJ Roberts JM Misore A Muga R Oloo AJ Steketee RW Efficacy of sulfadoxine-pyrimethamine for prevention of placental malaria in an area of Kenya with a high prevalence of malaria and human immunodeficiency virus infection Am J Trop Med Hyg 1998 59 813 822 9840604 10.4269/ajtmh.1998.59.813

[B76] Filler SJ Kazembe P Thigpen M Macheso A Parise ME Newman RD Steketee RW Hamel M Randomized trial of 2-dose versus monthly sulfadoxine-pyrimethamine intermittent preventive treatment for malaria in HIV-positive and HIV-negative pregnant women in Malawi J Infect Dis 2006 194 286 293 10.1086/505080 16826475

[B77] Hamer DH Mwanakasale V MacLeod WB Chalwe V Mukwamataba D Champo D Mwananyanda L Chilengi R Mubikayi L Mulele CK Mulenga M Thea DM Gill CJ Two-dose versus monthly intermittent preventive treatment of malaria with sulfadoxine-pyrimethamine in hiv-seropositive pregnant Zambian Women J Infect Dis 2007 196 11 1585 1594 10.1086/522142 18008241

[B78] Miller KD Lobel HO Satriale RF Kuritsky JN Stern R Campbell CC Severe cutaneous reactions among American travelers using pyrimethamine-sulfadoxine (fansidar(r)) for malaria prophylaxis Am J Trop Med Hyg 1986 35 451 458 2939735 10.4269/ajtmh.1986.35.451

[B79] Hellgren U Rombo L Berg B Carlson J Wiholm BE Adverse reactions to sulphadoxine-pyrimethamine in Swedish travellers: implications for prophylaxis BMJ 1987 295 365 366 2958104 10.1136/bmj.295.6594.365-a PMC1247216

[B80] Phillips-Howard P West LJ Serious adverse drug reactions to pyrimethamine-sulphadoxine, pyrimethamine-dapsone and to amodiaquine in Britain J R Soc Med 1990 83 82 85 2138674 10.1177/014107689008300208 PMC1292502

[B81] Gimnig JE MacArthur JR M'Bang'Ombe M Kramer MH Chizani N Stern RS Mkandala C Newman RD Steketee RW Campbell CH Severe cutaneous reactions to sulfadoxine-pyrimethamine and trimethoprim-sulfamethoxazole in Blantyre District, Malawi Am J Trop Med Hyg 2006 74 738 743 16687672

[B82] Luntamo M Kulmala T Mbewe B Cheung YB Maleta K Ashorn P Effect of Repeated treatment of pregnant women with sulfadoxine-pyrimethamine and azithromycin on preterm delivery in Malawi: a randomized controlled trial Am J Trop Med Hyg 2010 83 1212 1220 10.4269/ajtmh.2010.10-0264 21118924 PMC2990034

[B83] Khoo K The treatment of malaria in glucose-6-phosphate dehydrogenase deficient patients in Sabah Ann Trop Med Parasitol 1981 75 591 595 7325735 10.1080/00034983.1981.11687489

[B84] Cavenee MR Farris JR Spalding TR Barnes DL Castaneda YS Wendel GD Jr Treatment of gonorrhea in pregnancy Obstet Gynecol 1993 81 33 38 8416458

[B85] Adelson M Graves WL Osborne NG Treatment of urinary infections in pregnancy using single versus 10-day dosing J Natl Med Assoc 1992 84 73 75 1602504 PMC2637714

[B86] Brown C Saffan BD Howard CM Preedy JR The Renal clearance of endogenous estrogens in late pregnancy J Clin Invest 1964 43 295 303 10.1172/JCI104914 14164476 PMC289523

[B87] Lee FI Loeffler FE Gout and pregnancy J Obstet Gynaecol Br Emp 1962 69 2 299 304 10.1111/j.1471-0528.1962.tb00046.x 14463488

[B88] Weingold AB Gout and Pregnancy Obstet Gynecol 1960 16 3 309 313 13843594

[B89] Schackis RC Hyperuricaemia and preeclampsia: is there a pathogenic link? Med Hypotheses 2004 63 239 244 10.1016/j.mehy.2004.02.018 15236782

[B90] Czaczkes W Ullmann TD Sadowsky E Plasma uric acid levels, uric acid excretion, and response to probenecid in toxemia of pregnancy J Lab Clin Med 1958 51 224 229 13514228

[B91] Hernandez-Diaz S Werler MM Walker AM Mitchell AA Folic acid antagonists during pregnancy and the risk of birth defects NEJM 2000 343 1608 1614 10.1056/NEJM200011303432204 11096168

[B92] Sweet D Organic anion transporter (Slc22a) family members as mediators of toxicity Toxicol Appl Pharmacol 2005 204 198 215 10.1016/j.taap.2004.10.016 15845414

[B93] Anzai N Kanai Y Endou H Organic anion transporter family: current knowledge J Pharmacol Sci 2006 100 411 426 10.1254/jphs.CRJ06006X 16799257

[B94] Cundy KC Petty BG Flaherty J Fisher PE Polis MA Wachsman M Lietman PS Lalezari JP Hitchcock MJ Jaffe HS Clinical pharmacokinetics of cidofovir in human immunodeficiency virus- infected patients Antimicrob Agents Chemother 1995 39 1247 1252 7574510 10.1128/aac.39.6.1247 PMC162721

[B95] Coleman M Edwards G Mihaly GW Howells RE Breckenridge AM High-performance liquid chromatographic method for the determination of pyrimethamine and its 3-N-oxide metabolite in biological fluids J Chromatogr 1984 308 363 369 6746829 10.1016/s0021-9673(01)87569-5

[B96] Dua V Gupta NC Sethi P Edwards G Dash AP High-performance liquid chromatographic assay for the determination of sulfadoxine and N-acetyl sulfadoxine in plasma from patients infected with sensitive and resistant *Plasmodium falciparu *malaria J Chromatogr B Analyt Technol Biomed Life Sci 2007 860 160 165 10.1016/j.jchromb.2007.10.016 17997367

[B97] World Health Organization World Malaria Report 2009 2009 Geneva: World Health Organization

[B98] World Health Organization. Malaria in Pregnancy Working Group Minutes of the 8th Strategic Planning Meeting 2007 Geneva: World Health Organization

